# Impact of brain natriuretic peptide reduction on the worsening renal function in patients with acute heart failure

**DOI:** 10.1371/journal.pone.0235493

**Published:** 2020-06-26

**Authors:** Kenji Yoshioka, Yuya Matsue, Takahiro Okumura, Keisuke Kida, Shogo Oishi, Eiichi Akiyama, Satoshi Suzuki, Masayoshi Yamamoto, Akira Mizukami, Shunsuke Kuroda, Nobuyuki Kagiyama, Tetsuo Yamaguchi, Tetsuo Sasano, Akihiko Matsumura, Takeshi Kitai

**Affiliations:** 1 Department of Cardiology, Kameda Medical Center, Chiba, Japan; 2 Department of Cardiovascular Medicine, Tokyo Medical and Dental University, Tokyo, Japan; 3 Department of Cardiovascular Medicine, Juntendo University Graduate School of Medicine, Tokyo, Japan; 4 Cardiovascular Respiratory Sleep Medicine, Juntendo University Graduate School of Medicine, Tokyo, Japan; 5 Department of Cardiology, Nagoya University Graduate School of Medicine, Nagoya, Japan; 6 Department of Pharmacology, St. Marianna University School of Medicine, Kawasaki, Japan; 7 Department of Cardiology, Himeji Cardiovascular Center, Hyogo, Japan; 8 Division of Cardiology, Yokohama City University Medical Center, Kanagawa, Japan; 9 Department of Cardiovascular Medicine, Fukushima Medical University, Fukushima, Japan; 10 Cardiovascular Division, Institute of Clinical Medicine, Graduate School of Comprehensive Human Sciences, University of Tsukuba, Ibaraki, Japan; 11 Heart and Vascular Institute, Cleveland Clinic, Cleveland, OH, United States of America; 12 Department of Cardiology, The Sakakibara Heart Institute of Okayama, Okayama, Japan; 13 Department of Cardiology, Cardiovascular Center, Toranomon Hospital, Tokyo, Japan; 14 Department of Cardiovascular Medicine, Kobe City Medical Center General Hospital, Kobe, Japan; Scuola Superiore Sant'Anna, ITALY

## Abstract

**Aims:**

The prognostic impact of worsening renal function (WRF) in patients with acute heart failure (AHF) remains under debate. Successful decongestion might offset the negative impact of WRF, but little is known about indicators of successful decongestion in the very acute phase of AHF. We hypothesized that decongestion as evaluated by the percent reduction in brain natriuretic peptide (BNP) could identify relevant prognostic implications of WRF in the very acute phase of AHF.

**Methods and results:**

Data on 907 consecutive hospitalized patients with AHF in the REALITY-AHF study (age: 78±12 years; 55.1% male) were analyzed. Creatinine and BNP were measured at baseline and 48 hours from admission. WRF was defined as an increase in creatinine >0.3 mg at 48 hours from admission. The primary endpoint was 1-year all-cause mortality. Patients were divided into four groups according to the presence/absence of WRF and a BNP reduction higher/lower than the median: no-WRF/higher-BNP-reduction (n = 390), no-WRF/lower-BNP-reduction (n = 397), WRF/higher-BNP-reduction (n = 63), and WRF/lower-BNP-reduction groups (n = 57). Kaplan-Meier curve analysis showed that the WRF/lower-BNP-reduction group had a worse prognosis than the other groups. In a Cox regression analysis, only the WRF/lower-BNP-reduction group had higher mortality compared to the no-WRF/higher-BNP-reduction group (hazard ratio: 3.34, p<0.001).

**Conclusion:**

In the very acute phase of AHF, BNP reduction may aid in identifying relevant prognostic significance of WRF.

## Introduction

Accumulating evidences demonstrate the importance of treatment during the very acute phase in acute heart failure (AHF). Decongestion with intravenous loop diuretics is the mainstay treatment for AHF, as congestion is one of the main reasons for heart failure admission [1–4]. However, the use of loop diuretics causes worsening renal function (WRF), which has been reported to be associated with a poor prognosis in patients with AHF [5]. This association, however, does not always hold, as several recent studies have shown that the prognostic impact of WRF varies according to the clinical context in which it occurs [6–8]. More specifically, WRF occurring during successful decongestive treatment is not associated with a poor prognosis, whereas WRF occurring during an unfavorable clinical course is associated with a poor prognosis [6–8]. Distinguishing these two phenotypes of WRF is clinically relevant, as the subsequent treatment could differ. Therefore, it is critically important to understand the extent to which ongoing decongestive treatment is effective. A rational (bio)marker that provides such information is yet to be developed; however, the brain natriuretic peptide (BNP) level has been widely used as a marker of congestion, mainly because of its strong association with prognosis in patients with heart failure [9,10]. Especially in the very acute phase of AHF treatment, dynamic changes in the intravascular volume due to aggressive diuresis might provoke changes in both BNP and creatinine levels. However, very few studies have investigated the prognostic interaction between the change in BNP level and WRF during treatment in very acute phase AHF. We, therefore, hypothesized that combining information regarding changes in BNP and creatinine levels during the acute phase may enable the risk stratification of patients with AHF and WRF.

## Methods

### Study participants

The present study utilized data from the REALITY-AHF (Registry Focused on Very Early Presentation and Treatment in Emergency Department of Acute Heart Failure), a prospective multicenter registry focused on presentation and treatment during the very early phase of AHF hospitalization. Details regarding the study design have been published elsewhere [4,11–16]. Briefly, consecutive patients with AHF aged ≥20 years who were hospitalized via the emergency department (ED) at 20 hospitals in Japan were enrolled. The diagnosis of AHF was determined by an attending physician at each site, using the Framingham criteria [17]. All patients measured BNP or NT-proBNP at admission and those with BNP <100 ng/L or NT-proBNP <300 ng/L were excluded because of uncertainty in the diagnosis according to the guidelines [18]. Detailed inclusion/exclusion criteria and other study information were published in the publicly available University Hospital Information Network (UMIN-CTR, unique identifier: UMIN000014105) before the first patient was enrolled. All participants were informed about their participation in the study and it was explained that they were free to opt out of participation at any time. Written informed consent was not required under Japanese law due to the observational nature of the study. The study protocol was conducted in compliance with the Declaration of Helsinki. Study details, including the method of obtaining informed consent from participants, were first approved by Kameda Medical Center, Clinical Research Committee and subsequently approved by the ethical committee of each participating hospital before patient enrolment.

### Data collection

Baseline data, including physical findings, echocardiography, and laboratory tests were collected from the ED. Creatinine and BNP levels were evaluated at baseline and 48 hours of admission, and the change in these two parameters was calculated. All creatinine measurements were performed using enzymatic assay. The change in the BNP level was reported as the percent BNP reduction (i.e. positive and negative values indicated reduced and increased BNP, respectively). WRF was defined as an increase in creatinine ≥0.3 mg/dL from baseline (evaluated at ED arrival) to 48 hours of admission [19,20]. Patients were divided into four groups according to the presence/absence of WRF and higher/lower percent BNP reduction relative to the median (i.e. percent BNP reduction equal to or higher than the median vs. percent BNP reduction lower than the median). Patients with missing data for either creatinine or BNP at 48 hours of admission were grouped using creatinine and BNP at 24 hours. We excluded patients with missing data for either creatinine or BNP at baseline, as well as those who did not undergo the simultaneous measurement of creatinine and BNP at either 24 or 48 hours of admission. We did not include those with available data on only NT-proBNP but not BNP for consistency. All patients were prospectively followed up for 1 year after discharge, and the primary endpoint was all-cause mortality.

### Statistical analysis

Data were presented as mean ± standard deviation or median [1st–3rd quartile] for continuous variables, and as frequency (%) for categorical variables. One-way analysis of variance or the Kruskal-Wallis test was used to compare continuous variables. The χ^2^ or Fisher’s exact test was used to compare categorical variables. When necessary, variables were transformed for further analyses. Event-free survival curves were constructed using the Kaplan-Meier survival method and compared using log-rank statistics. A multivariable Cox regression analysis was performed, adjusting for the following variables, which were considered as preexisting and known prognostic factors: age, sex, New York Heart Association functional class, systolic blood pressure, heart rate, and history of heart failure. The analysis further adjusted for history of diabetes, left ventricular ejection fraction, prescription for a beta blocker, prescription for an angiotensin inhibitor or angiotensin II receptor blocker at admission, hemoglobin, serum sodium, serum creatinine, blood urea nitrogen (BUN), BNP, and C-reactive protein [11]. Multiple imputation was performed to account for missing covariate data. Twenty datasets were created using a chained-equations procedure [21]. Parameter estimates were obtained for each dataset and subsequently combined to produce an integrated result using the method described by Barnard and Rubin [22].

All statistical analyses were performed using R software (The R Foundation for Statistical Computing, Vienna, Austria). In all analyses, a two-tailed p-value <0.05 was considered to indicate statistical significance.

## Results

Among the 1,682 patients enrolled in the REALITY-AHF, 8 patients who died within 48 hours of admission and 61 patients with missing creatinine data at both 24 and 48 hours of admission were excluded. Further, 688 patients with missing BNP levels at 24 and 48 hours of admission and 18 patients who did not have creatinine and BNP levels simultaneously obtained at either 24 or 48 hours were excluded; 907 patients were finally analyzed. Characteristics were compared between included and excluded patients (S1 Table). Excluded patients had lower diastolic blood pressure, lower left ventricular ejection fraction, and higher prescription of mineralocorticoid receptor antagonists, as well as lower hemoglobin level than did included patients. No other statistically significant differences were found.

The mean age of the analyzed patients was 78±12 years and 55% were male. The mean left ventricular ejection fraction (measured before discharge) was 48%±16%, and 45% of the patients had heart failure with a preserved ejection fraction (defined as a left ventricular ejection fraction ≥50%).

According to the definition of WRF described in the Methods section, 120 (13.2%) patients were defined as having WRF. The BNP level significantly decreased from a mean level of 745 (interquartile range [IQR]: 431–1312) pg/mL at baseline to a mean level of 400 (IQR: 192–765) pg/mL at 48 hours (Wilcoxon signed-rank test: p<0.001). The median percent BNP reduction within 48 hours was 61% (IQR: 37–78%).

The no-WRF/higher-BNP-reduction group comprised 390 patients; the no-WRF/lower-BNP-reduction group comprised 397 patients; the WRF/higher-BNP-reduction group comprised 63 patients; and the WRF/lower-BNP-reduction group comprised 57 patients. Table 1 shows the baseline characteristics according to group. Overall, patients with WRF were older and more likely to have high blood pressure, a history of hypertension, and be on diuretics at admission than patients without WRF. As age increases, patients with WRF had higher creatinine and blood urea nitrogen levels and lower estimated glomerular filtration rate (eGFR) at discharge than those without WRF. In addition, patients with WRF had higher white blood cell count, lower hemoglobin, worse renal function, and higher BNP levels than those without WRF. On the other hand, patients with a percent BNP reduction equaled or above the median within 48 hours were more likely to have higher blood pressure and be on a beta-blocker at admission than those with a percent BNP reduction less than the median.

**Table 1:** Patient characteristics.

Variables	No WRF/less BNP reduction	No WRF/more BNP reduction	WRF/less BNP reduction	WRF/more BNP reduction	P-value
	n = 397	n = 390	n = 57	n = 63	
Age (years)	79±12	76±13	80±11	80±12	0.004
Male gender (%)	225 (56.7)	217 (55.6)	25 (43.9)	33 (52.4)	0.315
Systolic blood pressure (mmHg)	142±33	154±34	150±35	171±37	<0.001
Diastolic blood pressure (mmHg)	81±24	89±25	81±23	93±24	<0.001
Heart rate (bpm)	96±29	99±30	98±25	96±23	0.349
ECG rhythm (%)					0.646
Sinus	200 (50.4)	207 (53.1)	28 (50.0)	40 (63.5)	
Atrial fibrillation	155 (39.0)	142 (36.4)	22 (39.3)	19 (30.2)	
Others	42 (10.6)	41 (10.5)	6 (10.7)	4 (6.3)	
LVEF measured at emergency department (%)					0.389
<35%	126 (32.6)	151 (39.7)	21 (38.9)	19 (31.1)	
35–50%	111 (28.8)	106 (27.9)	14 (25.9)	16 (26.2)	
>50%	149 (38.6)	123 (32.4)	19 (35.2)	26 (42.6)	
Comorbidities (%)					
History of Heart Failure	212 (53.4)	185 (47.4)	30 (52.6)	34 (54.0)	0.366
Hypertension	268 (67.5)	263 (67.4)	45 (78.9)	53 (84.1)	0.017
Diabetes mellitus	165 (41.6)	119 (30.5)	23 (40.4)	24 (38.1)	0.013
Coronary artery disease	121 (30.5)	101 (25.9)	21 (36.8)	21 (33.3)	0.212
Medication at admission (%)					
Loop diuretics	229 (58.4)	179 (46.3)	30 (52.6)	36 (57.1)	0.007
ACE-I	54 (13.6)	68 (17.4)	6 (10.5)	12 (19.0)	0.272
ARB	132 (33.2)	108 (27.7)	18 (31.6)	28 (44.4)	0.045
Beta blocker	150 (38.3)	179 (45.9)	23 (40.4)	36 (57.1)	0.017
Aldosterone blocker	72 (18.1)	86 (22.1)	10 (17.5)	10 (15.9)	0.435
Laboratory data					
White blood cell count (/μl)	7100 [5400, 9500]	7700 [5900, 10200]	9000 [6800, 11600]	8800 [6450, 11550]	<0.001
Hemoglobin (g/dL)	11.6±2.3	12.3±2.3	10.8±2.2	11.6±2.2	<0.001
AST (IU/L)	32 [23, 48]	31 [24, 48]	30 [24, 40]	28 [21, 45]	0.504
ALT (IU/L)	21 [14, 35]	24 [16, 39]	18 [12, 32]	18 [13, 36]	0.012
Creatinine (mg/dL)	1.1 [0.8, 1.7]	1.0 [0.8, 1.4]	1.4 [0.9, 2.1]	1.5 [1.0, 1.8]	<0.001
Blood urea nitrogen (mg/dL)	25 [18, 37]	22 [17, 30]	29 [24, 42]	28 [22, 38]	<0.001
Sodium (mEq/L)	139±5	140±4	138±5	139±5	0.002
Glucose (mg/dL)	168±82	158±70	180±89	165±66	0.13
C-reactive protein (mg/dL)	0.58 [0.22, 2.11]	0.54 [0.17, 2.11]	1.14 [0.27, 2.57]	0.64 [0.25, 1.72]	0.181
BNP (pg/mL)	666 [403, 1186]	790 [464, 1393]	864 [456, 1563]	838 [435, 1472]	0.023

Values are expressed as mean ± SD, n (%), or median [interquartile range].

ECG, electrocardiogram; LVEF, left ventricular ejection fraction; ACEI, angiotensin converting enzyme inhibitor; ARB, angiotensin II receptor blocker; AST, aspartate transaminase; ALT, alanine transaminase; BNP, brain natriuretic peptide

**Fig 1:**
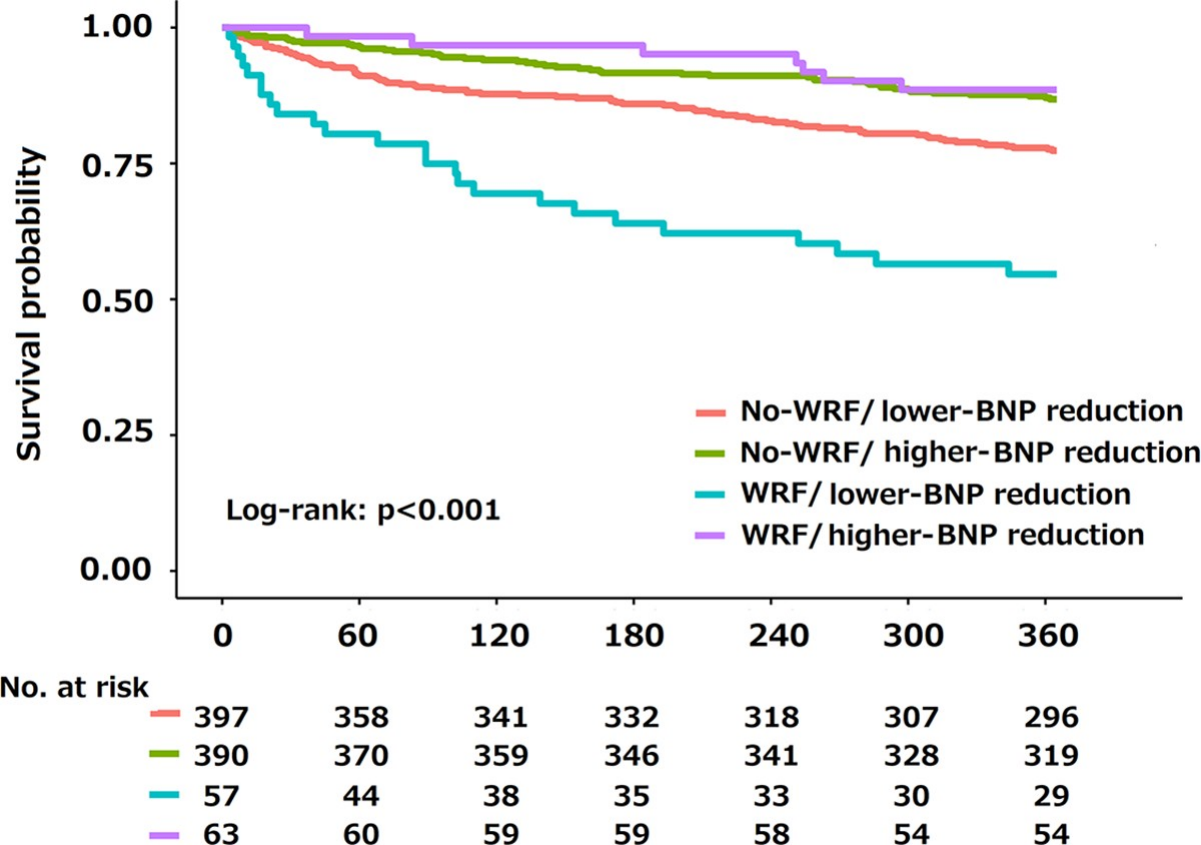
Association between worsening renal function (WRF), percent BNP reduction, and survival after discharge. Shown are the survival curves for groups according to WRF and change in percent BNP reduction at 48 hours of admission.

During 1-year of follow-up, there were 170 deaths, including 115 cardiovascular deaths (67.6%). Heart failure (67 deaths) was the most frequent cause of cardiovascular death (58% of all cardiovascular deaths). The Kaplan-Meier curve analysis showed that the WRF/lower-BNP-reduction group had a worse prognosis than did the other groups (Fig 1). Similarly, in the univariate/multivariate Cox regression analyses, membership in the WRF/lower-BNP-reduction group, but not membership in the WRF/higher-BNP-reduction or no-WRF/lower-BNP-reduction group, was associated with a higher mortality than that in the no-WRF/higher-BNP-reduction group (Table 2). Furthermore, there was a significant interaction between WRF and percent BNP reduction within 48 hours in terms of 1-year mortality, even in the multivariable model (p for the interaction = 0.029). To check if this association was affected by left ventricular ejection fraction, we calculated the *P* value for interaction and found it was not significant (*P* > 0.20).

**Table 2:** Univariate and multivariate Cox regression models.

Group	Univariate analysis			Multivariate analysis*			Multivariate analysis after multiple imputation*		
	HR	95% CI	P-value	HR	95% CI	P-value	HR	95% CI	P-value
No WRF/more reduction	1 (reference)			1 (reference)			1 (reference)		
No WRF/less reduction	1.83	1.29–2.56	<0.001	1.61	1.10–2.37	0.014	1.15	0.90–1.47	0.264
WRF/more reduction	0.84	0.38–1.87	0.683	0.77	0.34–1.75	0.537	1.00	0.64–1.58	0.986
WRF/less reduction	4.50	2.78–7.28	<0.001	3.34	1.95–5.73	<0.001	2.15	1.53–3.02	<0.001

*Adjustment was performed for age, gender, New York Heart Association functional class, systolic blood pressure, heart rate, history of heart failure, history of diabetes, left ventricular ejection fraction, prescription of beta blocker, prescription of angiotensin inhibitor or angiotensin II receptor blocker at admission, hemoglobin, serum sodium, serum creatinine, blood urea nitrogen (BUN) and BNP at baseline, as well as C-reactive protein.

We performed sensitivity analysis using multivariate Cox regression analysis with a different definition of WRF (>0.3 mg/dL and >25% increase in creatinine, 25% decrease in eGFR, and a 1.5-fold increase in creatinine from baseline) and 30% as the cut-off for the percent BNP reduction (S2 Table) [23]. Even though the point estimation of the hazard ratio for each group differed according to the definition of WRF, the results generally did not change. Moreover, we performed another sensitivity analysis by re-defining cut-off for WRF and percent BNP reduction using ROC curve analysis. The results of ROC analyses identified ≥0.16 mg/dL increase in creatinine and 29.9% decrease in percent BNP as the optimal cut-off to predict prognosis. The analysis using these values to group all cohort into four groups were not significantly different from the original results (S3 Table and S1 Fig).We used the creatinine and BNP at 24 hours of admission in cases with missing values at 48 hours, which might have impacted the study results. However, the same results were achieved when the patients with missing 48-hour data were excluded (S4 Table and S2 Fig)

## Discussion

The present study investigated the impact of the change in BNP level on the prognostic implication of WRF occurring within 48 hours of admission. In the present cohort, 13.2% experienced WRF and the median percent BNP reduction was 61%, with both parameters evaluated within 48 hours of admission. When patients were categorized into groups according to whether the percent BNP reduction was lower than the median, those in the WRF/lower-BNP-reduction group, but not those in the WRF/higher-BNP-reduction group, had high mortality. To the best of our knowledge, this is the first study to show that the percent change in BNP simultaneously assessed with creatinine can differentiate WRF with relevant prognosis from non-relevant WRF occurring in the very acute phase of AHF (i.e. within 48 hours of admission). Thus, the study results imply that creatinine changes occurring in the very acute phase should be interpreted in conjunction with the change in BNP in patients with AHF.

The prognosis of patients with AHF was initially thought to be worse in those with WRF than in those without WRF based on several observational studies [5]. However, subsequent studies have clearly shown that the clinical and prognostic implications of WRF were not always straightforward [6–8]. Moreover, it has been suggested that the timing of WRF assessment can alter its prognosis; in trials that defined WRF in the acute phase, WRF was not a simple prognostic marker. For example, in the post-hoc analysis of DOSE trial, which established protocol for aggressive diuretic dosing, the incidence of WRF at day 3 of AHF admission was not associated with worse outcomes. However, improving renal function at day 3 was an independent predictor of worse outcomes and persistent jugular venous distension at day 3 was less frequent in patients with WRF at day 3 [24]. Similarly, Valente et al. demonstrated that the incidence of WRF at day 7 of AHF admission was relatively high in the best diuretic response quintile, which had better long-term outcomes [25]. Although these findings implied that WRF accompanied with successful decongestion in the acute phase of AHF is not necessarily associated with a poor prognosis, consistent with the present findings, nevertheless, very few studies have focused on WRF occurring in the very acute phase of AHF. Metra et al. [26] utilized PROTECT study data to investigate whether the degree of congestion might impact the prognostic implication of WRF occurring in the very acute phase of AHF. They found that WRF (defined as a ≥0.3 mg/dL increase in creatinine from baseline) occurring at any time in the acute phase (study days 1 to 14) was consistently associated with a poor prognosis only if concomitant congestion existed. This finding is consistent with the present study results and reinforces the importance of congestion in terms of the prognostic relevance of WRF, even in the acute phase. Moreover, a focus on the very acute phase is particularly crucial given that most patients with AHF are treated with intensive diuretic therapy during this period, and the use of diuretics is one of the factors strongly associated with WRF. Thus, the present study results extended the current knowledge on the clinical and prognostic importance of WRF in patients with AHF.

Assessing the extent of decongestion correctly is essential in recognizing the prognostic meaning of WRF, but is also challenging. In previous studies, several indices, such as improvements in symptoms or physical examination findings, body weight reduction, net fluid loss, hemoconcentration, or BNP reduction, were used as markers of decongestion; however, a gold-standard marker of decongestion has not been established, especially in the very acute phase of AHF [6,8,27,28]. Among these indices, we chose to focus on BNP reduction as a marker of decongestion in the very acute phase of AHF. BNP is advantageous in terms of objectivity, reproducibility, and accessibility, and it is known as one of the best prognostic makers in patients with heart failure [9,10]. Indeed, we previously reported that the percent BNP reduction from admission to discharge is superior to the percent body weight reduction in terms of the risk stratification of patients with AHF, and is an independent predictor of worse outcomes [15]. Stolfo et al. [8] investigated the impact of the percent BNP reduction during admission on the prognostic implication of WRF in 122 patients with AHF, and showed that the prognosis of those with WRF was not necessarily poor, as long as a sufficient BNP reduction was achieved. However, the authors were not able to perform a multivariate analysis due to the small sample size and number of events. These previous studies support the evaluation of the magnitude of decongestion throughout admission, using the BNP reduction from baseline to discharge as a decongestion marker. However, we hypothesized that the change in BNP could also be a suitable index of decongestion in the very acute phase of AHF because, reportedly, BNP responds timely to a change in left ventricular filling pressure [29,30].

### Limitations

The present study has several limitations that should be acknowledged. This study did not comprise a predefined, post-hoc analysis of registry data; thus, the results should be interpreted cautiously. Additionally, a non-trivial number of patients was excluded because of missing BNP data at both 24 and 48 hours of admission. Although the baseline characteristics of the included and excluded patients were highly similar (with some exceptions) and a re-analysis after multiple imputation achieved the same results, the present study results and conclusion might have been impacted by this exclusion. Furthermore, we used the creatinine and BNP at 24 hours of admission in cases with missing values at 48 hours, which might have influenced the study results. However, the same results were achieved when the patients with missing 48-hour data were excluded (S4 Table and S2 Fig); therefore, we assumed that this convention did not influence the conclusion. As this study is a multicenter study, we did not use the same BNP assay in all participating institutions. This might have impacted our study results because it is well known that there are large systematic differences (up to 2 folds) between the methods used for BNP assay [31–33]; however, we did not use absolute change of BNP, but the variations (expressed as percent reduction compared to basal value) that mitigate this bias. Nevertheless, this point should be well acknowledged as a potential limitation. Finally, although there is no universally accepted definition of WRF, we defined WRF as an increase in creatinine ≥0.3 mg/dL from baseline; a similar issue existed for the cut-off of the percent BNP reduction achieved within 48 hours. Although the application of another definition of WRF, used in several previous studies, did not substantially change the results and supported our conclusion, the optimal definition of WRF and cut-off for percent BNP reduction achieved within 48 hours remain unclear and need to be established in future research.

In conclusion, the association between WRF occurring within 48 hours of admission and 1-year all-cause mortality in patients with AHF is significantly impacted by the percent BNP reduction achieved within this same period. The present study results suggest that BNP is a promising marker of decongestion, providing information regarding the prognostic relevance of WRF that cannot be achieved otherwise.

## Supporting information

S1 Fig Association between WRF, percent BNP reduction, and survival after discharge using the cut-off of ≥0.16 mg/dL increase in creatinine and 29.9% decrease in percent BNP based on ROC analyses. (DOCX)

S2 Fig Association between WRF, percent BNP reduction, and survival after discharge excluding those with missing 48-hour data on creatinine and BNP. (DOCX)

S1 Table Characteristics between included and excluded patients. (DOCX)

S2 Table Results of multivariable Cox regression analysis applying different definitions of WRF and cut-off for percentage reduction in BNP within 48 hours. (DOCX)

S3 Table Results of multivariable Cox regression analysis defining WRF (≥0.16 mg/dL increase in creatinine) and more/less reduction in BNP (≥29.9%) by ROC curve analyses. (DOCX)

S4 Table Results of multivariable Cox regression analysis excluding those with missing 48-hour data on creatinine and/or BNP (N = 798). (DOCX)
