# Supplementary material for: Impact of brain natriuretic peptide reduction on the worsening renal function in patients with acute heart failure
Source: PLoS One. 2020 Jun 26;15(6):e0235493. doi: 10.1371/journal.pone.0235493 (PMC7319326; doi:10.1371/journal.pone.0235493)
Supplement: S1 Fig — (DOCX) [file pone.0235493.s001.docx]

**S1 Fig. Association between WRF, percent BNP reduction, and survival after discharge using the cut-off of ≥0.16 mg/dL increase in creatinine and 29.9% decrease in percent BNP based on ROC analyses.**

**
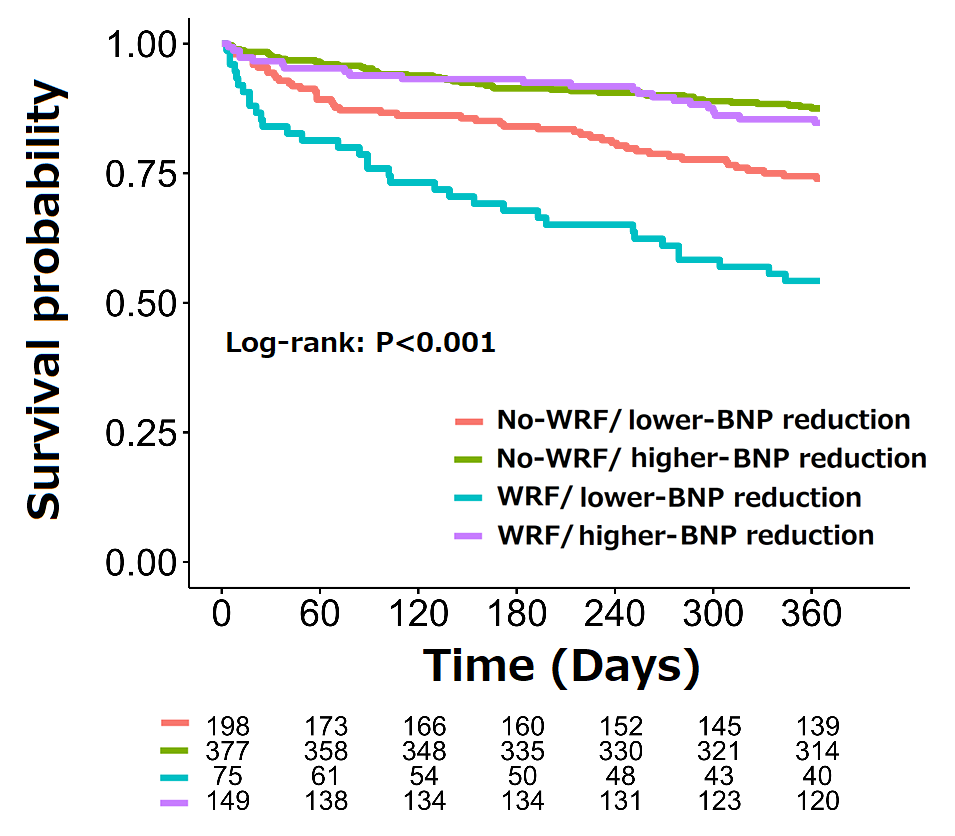
**
