# Supplementary material for: Impact of brain natriuretic peptide reduction on the worsening renal function in patients with acute heart failure
Source: PLoS One. 2020 Jun 26;15(6):e0235493. doi: 10.1371/journal.pone.0235493 (PMC7319326; doi:10.1371/journal.pone.0235493)

**Supplemental Figure 2. Association between WRF, percent BNP reduction, and survival after discharge excluding those with missing 48-hour data on creatinine and BNP.**


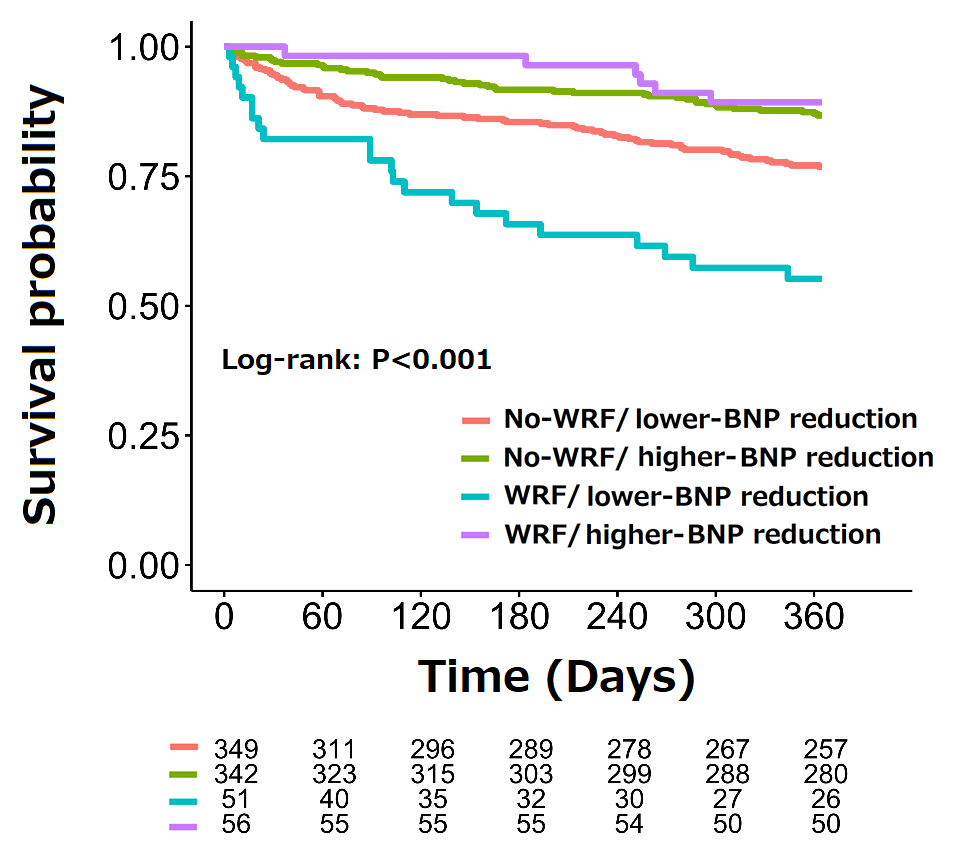

Supplement: S2 Fig — (DOCX) [file pone.0235493.s002.docx]
