# Supplementary material for: Impact of brain natriuretic peptide reduction on the worsening renal function in patients with acute heart failure
Source: PLoS One. 2020 Jun 26;15(6):e0235493. doi: 10.1371/journal.pone.0235493 (PMC7319326; doi:10.1371/journal.pone.0235493)
Supplement: S1 Table — (DOCX) [file pone.0235493.s003.docx]

**S1 Table. Characteristics between included and excluded patients.**

| Variables | Non-missing | Missing | P-value |
| --- | --- | --- | --- |
|  | n=907 | n=775 |  |
| Age (years) | 78±12 | 77±12 | 0.208 |
| Male gender (%) | 500 (55.1) | 433 (55.9) | 0.797 |
| Systolic blood pressure (mmHg) | 150±35 | 148±40 | 0.342 |
| Diastolic blood pressure (mmHg) | 85±25 | 82±25 | 0.014 |
| Heart rate (bpm) | 98±29 | 97±28 | 0.799 |
| ECG rhythm (%) |  |  | 0.154 |
| Sinus | 475 (52.4) | 438 (57.0) |  |
| AF | 338 (37.3) | 254 (33.0) |  |
| Others | 93 (10.3) | 77 (10.0) |  |
| LVEF measured at emergency department (%) |  |  | 0.040 |
| <35% | 317 (36.0) | 277 (39.9) |  |
| 35-50% | 247 (28.0) | 210 (30.2) |  |
| >50% | 317 (36.0) | 208 (29.9) |  |
| Comorbidities (%) |  |  |  |
| History of Heart Failure | 461 (50.8) | 393 (50.8) | >0.99 |
| Hypertension | 629 (69.3) | 501 (64.8) | 0.054 |
| Diabetes mellitus | 331 (36.5) | 289 (37.4) | 0.728 |
| Coronary artery disease | 264 (29.1) | 245 (31.7) | 0.265 |
| Medication at admission (%) |  |  |  |
| Loop diuretics | 474 (52.7) | 377 (48.8) | 0.124 |
| ACE-I | 140 (15.4) | 145 (18.8) | 0.075 |
| ARB | 286 (31.5) | 229 (29.8) | 0.470 |
| Beta blocker | 388 (43.0) | 334 (43.4) | 0.921 |
| Aldosterone blocker | 178 (19.6) | 193 (25.0) | 0.010 |
| Laboratory data |  |  |  |
| White blood cell count (/µl) | 7600 [5800, 10210] | 7600 [5800, 9975] | 0.760 |
| Hemoglobin (g/dL) | 11.9±2.3 | 11.6±2.3 | 0.013 |
| AST (IU/L) | 31 [23, 48] | 32 [22, 46] | 0.476 |
| ALT (IU/L) | 22 [14, 37] | 21 [13, 35] | 0.279 |
| Creatinine (mg/dL) | 1.1 [0.8, 1.6] | 1.1 [0.8, 1.6] | 0.665 |
| Blood urea nitrogen (mg/dL) | 25 [18, 35] | 25 [18, 37] | 0.077 |
| Sodium (mEq/L) | 139±5 | 139±5 | 0.047 |
| Glucose (mg/dL) | 164±77 | 170±81 | 0.151 |
| C-reactive protein (mg/dL) | 0.59 [0.20, 2.18] | 0.70 [0.21, 2.03] | 0.607 |
| BNP (pg/mL) | 745 [432, 1312] | 744 [467, 1425] | 0.334 |

Values are mean ± SD, n (%), or median [interquartile range].

ECG, electrocardiogram; LVEF, left ventricular ejection fraction; ACEI, angiotensin converting enzyme inhibitor; ARB, angiotensin II receptor blocker; AST, aspartate transaminase; ALT, alanine transaminase; BNP, brain natriuretic peptide
