# Supplementary material for: Impact of brain natriuretic peptide reduction on the worsening renal function in patients with acute heart failure
Source: PLoS One. 2020 Jun 26;15(6):e0235493. doi: 10.1371/journal.pone.0235493 (PMC7319326; doi:10.1371/journal.pone.0235493)
Supplement: S2 Table — (DOCX) [file pone.0235493.s004.docx]

**S2 Table. Results of multivariable Cox regression analysis applying several different definitions of WRF and cut-off for percentage reduction in BNP within 48 hours.**

| Group | WRF: >0.3 mg/dL and >25% increase in creatinine | | |  | WRF: >25% decrease in eGFR | | |  | WRF: 1.5-fold increase in creatinine | | |  | BNP: achieve >30% reduction or not | | |
| --- | --- | --- | --- | --- | --- | --- | --- | --- | --- | --- | --- | --- | --- | --- | --- |
|  | HR | 95% CI | P-value |  | HR | 95% CI | P-value |  | HR | 95% CI | P-value |  | HR | 95% CI | P-value |
| No WRF/more reduction | 1 (reference) | | |  | 1 (reference) | | |  | 1 (reference) | | |  | 1 (reference) | | |
| No WRF/less reduction | 1.66 | 1.14-2.43 | 0.009 |  | 1.71 | 1.16-2.51 | 0.006 |  | 1.84 | 1.28-2.65 | 0.001 |  | 1.96 | 1.36-2.82 | <0.001 |
| WRF/more reduction | 1.06 | 0.45-2.51 | 0.894 |  | 1.33 | 0.62-2.85 | 0.472 |  | 2.34 | 0.70-7.74 | 0.166 |  | 1.01 | 0.51-2.02 | 0.975 |
| WRF/less reduction | 4.84 | 2.71-8.65 | <0.001 |  | 4.32 | 2.47-7.57 | <0.001 |  | 5.59 | 2.58-12.1 | 0.472 |  | 3.64 | 2.11-6.29 | <0.001 |

In all models, adjustment was performed for age, gender, New York Heart Association functional class, systolic blood pressure, heart rate, history of heart failure, history of diabetes, left ventricular ejection fraction, prescription of beta blocker, prescription of angiotensin inhibitor or angiotensin II receptor blocker at admission, hemoglobin, serum sodium, serum creatinine, blood urea nitrogen and BNP at baseline, and C-reactive protein.
