# Supplementary material for: Impact of brain natriuretic peptide reduction on the worsening renal function in patients with acute heart failure
Source: PLoS One. 2020 Jun 26;15(6):e0235493. doi: 10.1371/journal.pone.0235493 (PMC7319326; doi:10.1371/journal.pone.0235493)
Supplement: S4 Table — (DOCX) [file pone.0235493.s006.docx]

**S4 Table. Results of multivariable Cox regression analysis excluding those with missing 48-hour data on creatinine and/or BNP (N=798).**

| Group | Univariate analysis | | |  | Multivariate analysis | | |
| --- | --- | --- | --- | --- | --- | --- | --- |
|  | HR | 95% CI | P value |  | HR | 95% CI | P value |
| No WRF/more reduction | 1 (reference) | | |  | 1 (reference) | | |
| No WRF/less reduction | 1.87 | 1.30-2.71 | <0.001 |  | 1.67 | 1.11-2.51 | 0.014 |
| WRF/more reduction | 0.79 | 0.34-1.85 | 0.583 |  | 0.77 | 0.32-1.85 | 0.560 |
| WRF/less reduction | 4.35 | 2.61-7.27 | <0.001 |  | 3.10 | 1.74-5.53 | <0.001 |

Adjustment was performed for age, gender, New York Heart Association functional class, systolic blood pressure, heart rate, history of heart failure, history of diabetes, left ventricular ejection fraction, prescription of beta blocker, prescription of angiotensin inhibitor or angiotensin II receptor blocker at admission, hemoglobin, serum sodium, serum creatinine, blood urea nitrogen and BNP at baseline, and C-reactive protein.
